# Visible Laser Light Mediated Cancer Therapy via Photothermal Effect of Tannin-Stabilized Magnetic Iron Oxide Nanoparticles

**DOI:** 10.3390/nano13091456

**Published:** 2023-04-25

**Authors:** Nikesh Gupta, Chetna Gupta, Himadri B. Bohidar

**Affiliations:** 1 Special Centre for Nanosciences (SCNS), Jawaharlal Nehru University, New Delhi 110067, India; 2 Department of Chemistry, Hansraj College, University of Delhi, Delhi 110007, India; 3 School of Physical Sciences, Jawaharlal Nehru University, New Delhi 110067, India

**Keywords:** super-paramagnetic, iron oxide nanoparticles, visible light, photo-thermal effect, hyperthermia, cancer therapy, nanomedicine

## Abstract

Super-paramagnetic iron oxide nanoparticles (SPIONs/Fe_3_O_4_) were synthesized in aqueous medium under a nitrogen atmosphere. These particles were made water-dispersible by cladding them with tannic acid (TA). The synthesized nanoparticles were characterized for their size and surface charge using HRTEM and zetasizer. It was found that the size of the particles formed was around 15 nm with almost spherical morphology and negative surface charge. Vibrating sample magnetometer (VSM) data attributed a super-paramagnetic nature to these nanoparticles. The photo-thermal dynamics of these magnetite (Fe_3_O_4_) nanoparticles was characterized by exciting their dispersions with laser radiation in the visible region (635 nm). Remarkably, 17 min of laser irradiation of the dispersion raised its temperature by ~25 °C (25 to 49.8 °C), whereas for the solvent, it was limited to not more than 4 °C (after 60 min). Thus, the Fe_3_O_4_ nanoparticles generated localized hyperthermia for potential use in cancer therapy of tumor management. The photo-thermal dynamics of these nanoparticles was investigated *in-vitro* for cancer therapy, and it was clearly shown that cancer cell growth was inhibited, and considerable cellular damage occurred when cells were incubated with laser-activated magnetic nanoparticles. No noticeable innate toxicity of the nanoparticles was observed on cancer cell lines. The effectiveness of these nanoparticles was studied on several malignant cell lines, and an acceptable Fe_3_O_4_ concentration range was subsequently determined for generating substantial cell death by hyperthermia, but not inherent toxicity. Therefore, we concluded that this nano-system is effective and less time consuming for the treatment of malignant diseases such as cancer.

## 1. Introduction

Magnetic nanoparticles have various pharmaceutical applications, such as drug administration, cancer treatment, hyperthermia, magnetic separation, and magnetic resonance imaging (MRI) [1,2,3,4,5]. Novel synthetic techniques to manufacture customized nanoparticles with the capacity to rigorously regulate the structure of the magnetic core (such as size, shape, crystalline nature, and mono-dispersity) have been reported as a result of rapid breakthroughs in the field of nanotechnology [6,7,8,9,10,11]. Because of their unique magnetic characteristics, non-toxicity, and biocompatibility, SPIONs have been frequently employed in biomedical research. A fundamental problem in preclinical nanoparticle-mediated thermo-therapeutic research is the development of novel, multifunctional, more efficient, and safer therapies. Magnetic hyperthermia is generated via an alternating magnetic field (AMF) to oscillate the magnetic moment of an individual nanoparticle, converting magnetic energy into heat. Localized hyperthermia can be used to destroy malignant cells, either alone [12] or in combination with chemotherapy [13,14], or to increase the release of a drug to a specific site/tissue or cell [15,16,17].

The creation of nanostructures with integrated therapeutic properties is a viable preclinical strategy to cancer treatment [18,19,20]. Metallic [21] and magnetic nanoparticles [22] were among the promising agents in the early stages of nanoscale-based antitumoral thermotherapeutic probes. Normal tissues are less sensitive to a rise in temperature or heat as compared with cancer cells, thus hyperthermic tumor treatment has typically been given to the entire body or to superficial tissues [23]. Magnetic nanoparticles used to induce hyperthermia appear to have certain benefits because heat-generating nanoparticles may be manufactured in colloidal solutions that can be injected, avoiding the requirement for macroscopic implants. Hyperthermia-based photothermal therapy (PTT) might one day be used in clinics as a cancer treatment technique. It is mediated by metallic nanoparticles [24,25,26,27,28,29] or, possibly, semiconducting carbon nanotubes [30] or graphene [31] that can be triggered by near-infrared (NIR) light, which has low tissue absorption [32]. Magnetic iron oxide nanoparticles, on the other hand, have been examined in people following injection of nanoparticles directly into solid tumors for magnetic hyperthermia therapy of glioblastoma [33] and prostate cancer [34]. However, both the magnetic and photothermal techniques have some drawbacks. Some of these disadvantages include the fact that bystander tissues might get damaged owing to high levels of laser irradiation and some of the nanoparticles may be biopersistant and slightly toxic. Iron oxide nanoparticles, on the other hand, have previously been authorized for human use as MRI contrast agents [35]. The dipole relaxation generated by an alternating magnetic field causes magnetic hyperthermia of iron oxide nanoparticles. The heat produced by locally injected magnetic nanoparticles can precisely eliminate tumors while causing no harm to healthy cells. The AMF power and nanoparticle concentrations in the tumor may also readily alter the local temperature. Recently, the photothermal impact of Fe_3_O_4_/alumina [36] and Fe_3_O_4_/mSiO_2_ [37] core–shell nanoparticles on bacteria and KB (human epithelial carcinoma cells—known to be a subline of the ubiquitous KERATIN-forming tumor cell line HeLa) cancer cells were described. These have demonstrated good *in-vivo* biodegradability, and the iron ions released upon dissolution may be ingested by the body via a closely controlled physiological mechanism. Treatment with superparamagnetic nanoparticles is of special interest as it has far less adverse effects than chemotherapy or radiation. With the effective photothermal action of magnetic Fe_3_O_4_ nanoparticles, it may be feasible to therapeutically implement a hyperthermia cancer treatment utilizing visible light instead of AMF. Aqueous dispersion of magnetite nanoparticles is not highly stable for a longer duration at room temperature, and this might be a point of concern during *in-vivo* studies. Therefore, scientists are working with different surface modifications using biocompatible, non-toxic, and natural stabilizers such as chitosan (cationic/anionic), tannic acid, PEG, and so on, in order to have a greater stability both *in-vitro* and *in-vivo* [38,39,40]. Because of strong chelating interactions between catechol groups and metal ions, certain investigations have found that TA provides significant chemical flexibility, notably in metal ion complexation. Furthermore, these articles revealed the following outstanding TA characteristics: (a) the thickness of the TA layer may be readily changed by repeating the coating method, and (b) the many catechol groups provide a high reduction capacity to transform metal ions into metal oxide NPs without the use of an external reducing agent. Tannic acid consists of 10 galloyl groups, which allows it to function as a chelating and non-toxic coating material. These characteristics highlight TA’s suitability as a contender for drug delivery and protein immobilization applications [41,42]. Owing to the presence of multiple hydroxyl groups within the molecule, it can bind easily with the metal oxide and shows greater bonding and stability in water. Tannic-acid-coated iron oxide nanoparticles demonstrate negative zeta potential, which is a prerequisite for nanoparticles’ stability in suspension and biological environment [43]. Although positively charged formulations have a higher probability of cell absorption than negatively charged ones, they are more likely to be internalized by non-targeting cells. In contrast, neutral or low negative charge surface formulations, plasma protein adsorption, and nonspecific cellular interactions are at a minimum. As a result, lowering the zeta potential enhances the stability inside the body. Cancer cells are vulnerable to hyperthermia as temperatures over 43 °C for several minutes can cause apoptosis [44,45,46]. It is also believed that a higher temperature affects the cytoskeleton, cell membrane, and proteins involved in DNA damage repair, further destabilizing the cell and offsetting homeostasis, thereby making cancer cells more sensitive to chemotherapy [47,48,49]. Tumors are more vulnerable to hyperthermia than normal tissues because of their rapid cell division, low pH, increased hypoxia, and restricted temperature control due to poor fluid transport [50]. Most of the research articles related to magnetic-nanoparticle-based hyperthermia/PTT have shown the efficacy of SPION under the NIR or near NIR region. As per our understanding and literature survey, this is the first study to report the efficacy of tannic-acid-coated iron oxide nanoparticles for potential photo-thermal application under the visible region (635 nm). In this article, we have reported the synthesis of tannic-acid-stabilized superparamagnetic iron oxide nanoparticles for hyperthermia-based therapy in breast and lung cancer cell lines using continuous visible red light with a wavelength of 635 nm.

## 2. Materials and Methods

### 2.1. Materials

Iron salts—ferric chloride and ferrous chloride—were procured from Alfa Aesar. Ammonia and absolute ethanol were purchased from Rankem and Merck, respectively. MTT (3-(4,5-dimethylthiazol-2-yl)-2,5-diphenyltetrazolium bromide) was procured from Sigma Aldrich. Tannic acid (TA) was purchased from Thomas Baker. All the reagents were used as received without any further purification or modification. Milli Q water was used to prepared aqueous solutions. Nitrogen gas was obtained from Sigma Gases. All procedures were performed at room temperature unless otherwise stated.

### 2.2. Experimental Procedure

#### 2.2.1. Synthesis of Ultrasmall Superparamagnetic Iron Oxide (Fe_3_O_4_) Nanoparticles

The formulation was developed by co-precipitation of iron chloride salts with ammonia. Briefly, 70 mL of 2.2% solution of FeCl_3_.6H_2_O and 70 mL of 0.9% solution of FeCl_2_.4H_2_O were mixed with continuous stirring under nitrogen atmosphere at ~60 °C in a two-neck round-bottom flask. During vigorous stirring, 8.5 mL of liquid ammonia was added into the vortex of the iron salt solution. After stirring for 30 min at the desired temperature and atmosphere, black precipitates obtained were collected with the help of a magnet and washed three to four times with absolute alcohol (ethanol) followed by MQ water. The so obtained nanoparticles were then dispersed in an alcohol/water solution (1:10% (*v*/*v*)).

#### 2.2.2. Coating of Nanoparticles with Natural Polyphenols

The required amount of obtained magnetic nanoparticles was dispersed in 30 mL of MQ water and the solution was stirred for 30 min at RT. Once the particles are dispersed, 5 mg of pure tannic acid (or 5 mL of green tea leaves’ extract; 25% *w*/*w* of sample) was added and the solution was kept under stirring at RT for another 24 h. Then, the solution was centrifuged, or the particles were separated using a magnet, and the pellet thus obtained was washed three to four times with MQ water to remove excess/free tannic acid and used as such for physical characterizations and further applications.

### 2.3. Size and Surface Charge Determination

#### 2.3.1. High-Resolution Transmission Electron Microscopy (HRTEM)

A TECNAI G^2^-30U Twin equipment was used to perform HRTEM measurements. A drop of particles dispersed in MQ water was placed on TEM copper grids and the aqueous solution was progressively evaporated at RT.

#### 2.3.2. Dynamic Light Scattering (DLS)

The NICOMP^TM^ 380ZLS equipment was used for the measurements. The particle size was determined using the Stokes–Einstein equation d = kT/3πηD, where d is the particle diameter, k is the Boltzman’s constant, D is the translational diffusion coefficient, η is the viscosity of the liquid medium in which the particles are suspended, and T is the absolute temperature.

#### 2.3.3. Zeta Potential Measurement

The zeta potential measurement was performed in a Malvern Zetasizer Nano-ZS instrument using Malvern DTS 1070 cell.

### 2.4. Selected Area Electron Diffraction (SAED)/Electron Diffraction Spectroscopy (EDS)

SAED was measured using a TECNAI G^2^-30U Twin instrument. At room temperature, a drop of reasonably dispersed particles in MQ water or in a mixture of alcohol/water solution was deposited onto copper grids, and an image was taken after the solvent was evaporated.

### 2.5. Vibrating Sample Magnetometer (VSM) for Magnetization

To determine the magnetic nature of the developed formulation, 4 to 5 mg of powdered sample was wrapped in Teflon tape and analyzed at 300 K (instrument temperature range: 2 to 1050 K) using a vibrating sample magnetometer (Model 3473-70) with an electromagnet amplifier (CREST Performance CPX 900 power amplifier Instrument).

### 2.6. Photothermal Measurements

#### 2.6.1. Rise in Temperature Using a Visible Red Light 635 nm Laser (Temperature-Dependent Profile)

To monitor the temperature change in solution containing Fe_3_O_4_ nanoparticles, the sample was irradiated with visible red laser light (635 nm diode laser and a power density of 38 mWcm^−2^). For a typical experiment, aqueous solutions with four different concentrations of Fe_3_O_4_ were placed into a transparent 5 mL glass vial. From the top of the vial, a digital temperature measurement probe/thermocouple was inserted to measure the variation in temperature in real time and laser light was used to irradiate the solution for 20 min. For the control, the rise in temperature of MQ water/PBS (without nanoparticles) irradiated by the same laser was measured for 60 min.

#### 2.6.2. Heat Map Cycle

To check the hyperthermia efficiency of the prepared formulation, the magnetic nanoparticles were irradiated with the continuous 635 nm red light for 20 min (ON/OFF cycles) and a heat map was generated using a temperature thermocouple time-dependent study.

### 2.7. Effect of Heat/Temperature on Polythene Bag

The Fe_3_O_4_ nanoparticles were separated from aqueous solutions and freeze-dried/lyophilized. The known amount of lyophilized powder (5.24 mg) was kept inside one of the corners of a polyethylene bag. A laser with a continuous wavelength of 635 nm was used to irradiate the lyophilized Fe_3_O_4_ powder, which was kept inside one corner of the polythene plastic bag at room temperature. In the case of the control, the sample bag without any particles was irradiated with the experimental laser. Burning and/or damage in the plastic bag due to generation of heat was observed/monitored.

### 2.8. In-Vitro Cell Culture Experiments

#### 2.8.1. MTT (3-(4,5-Dimethylthiazole-2yl)-2,5-Diphenyltetrazolium Bromide) Assay

The cytotoxicity of SPIONs, laser-treated SPIONs (SPION_laser@635nm_), and laser-treated SPIONs in combination with an external magnetic field (_EMF_SPIONs_laser@635nm_) were measured by MTT assay. Three different cancer cell lines (such as MCF-7, A549, and MDA-MB231) were maintained as per the provided protocol. Briefly, cells were seeded in a 96-well flat bottom plate at a density of 5000 cells/well in 100 μL of complete culture media (500 mL media containing 10% fetal bovine serum (FBS), 1% sodium pyruvate, and 1% penicillin streptomycin (Pen-Strep)). After 16 h of incubation, the media was aspirated and the cell monolayer was treated with different concentrations of SPIONs (0.5 mg/mL, 1 mg/mL, 5 mg/mL, and 10 mg/mL), and selected wells were simultaneously irradiated with visible red laser of 635 nm for 20 min in the presence and absence of an external magnetic field. After the pre-set irradiation time point, the cells were incubated in a humidified environment of 5% CO_2_ at 37 °C for 2 h. Then, the treatment groups’ media were replaced with fresh media and the plates were incubated for an additional 24 h. After the mentioned incubation time period, 20 μL of MTT (5 mg/mL) was added in each well and, after two hours, the precipitates were dissolved in 100 μL DMSO and quantified by measuring the absorbance at 570 nm using a micro plate reader. Cells cultured in media only or without SPIONs were taken as the negative control.

#### 2.8.2. Cell Count Analysis

The efficacy of magnetic nanoparticles was also confirmed using a cell count analysis experiment. Briefly, 1 × 10^4^ cells (MCF-7) per well were plated in a 24-well tissue culture plate and left overnight inside the incubator at 37 °C. The next day, the wells were treated with the above formulation in the presence and absence of the laser and the total number of viable cells was counted after 24 h.

#### 2.8.3. Wound Healing/Scratch Assay

The cells at a density of 1 × 10^5^ cells/well (A549) were seeded in a 24-well culture plate. After the confirmation of a confluent monolayer formation, a scratch in each well was gently made using a sterile 200 μL micropipette tip. The magnetic-nanoparticle-treated wells were irradiated with a laser light in the presence and absence of an external magnetic field and the wound healing/scratch closure in all the experimental wells compared with the control was monitored over a period of 48 h or till its closure. The untreated wells or the wells with medium alone were considered as the negative control. The analysis of the images was carried out using ImageJ software and % closure was then calculated as per the provided instructions.

## 3. Results and Discussion

**Figure 1:**
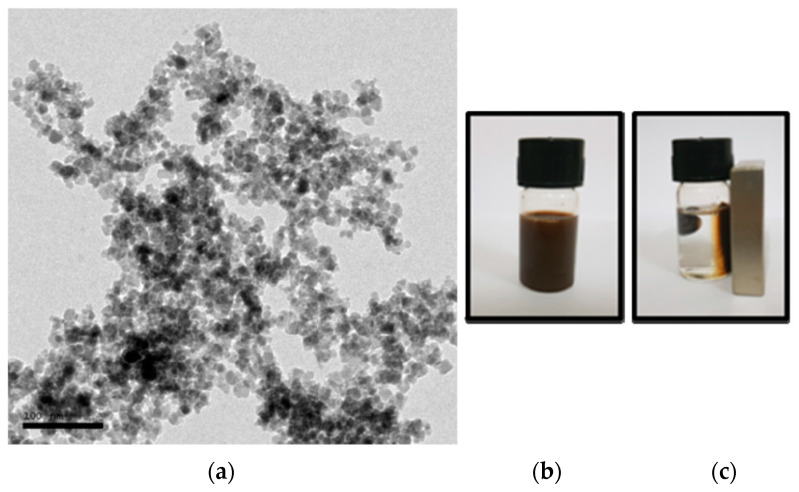
(**a**) High-resolution transmission electron microscopic (HRTEM) image of iron oxide nanoparticles with an average size of 15 nm. Scale bar represents 100 nm. (**b**) Digital image of iron oxide nanoparticles dispersed in MQ water. (**c**) Digital image of iron oxide nanoparticles totally attracted on the side of glass wall in the presence of an external magnetic field.

**Figure 2:**
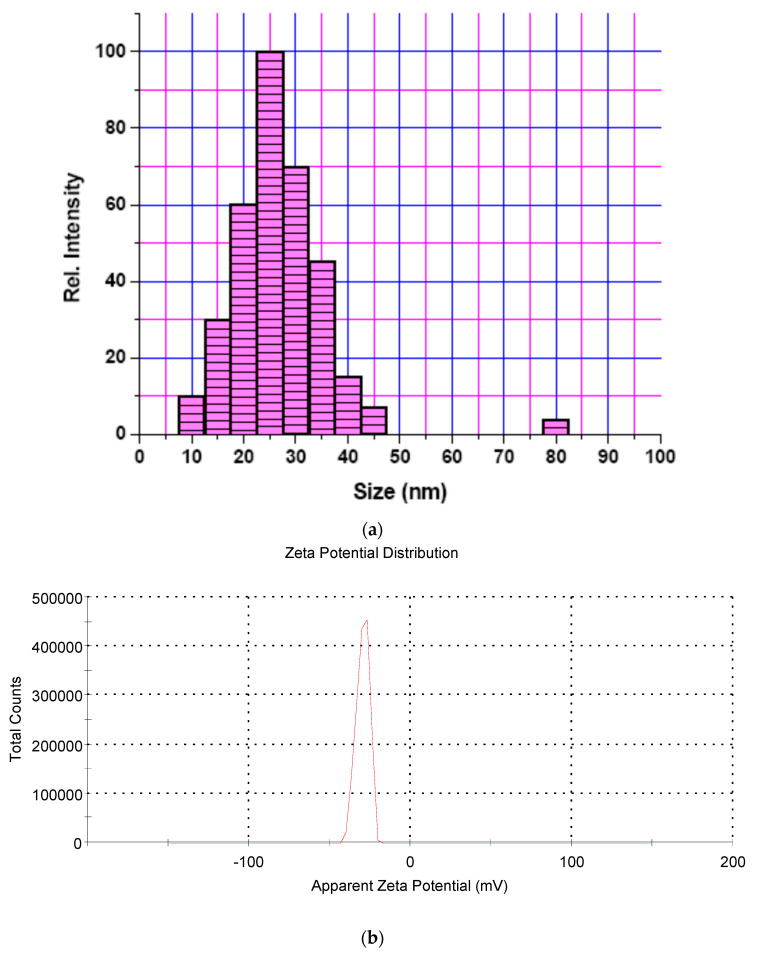
Size determination and zeta potential measurement: (**a**) Dynamic light scattering (DLS) pattern of iron oxide nanoparticles showing maximum distribution at 25 nm. The data represent the average of three individual runs. (**b**) Zeta potential distribution curve of the synthesized magnetic iron oxide nanoparticles showing a negative surface charge.

**Figure 3:**
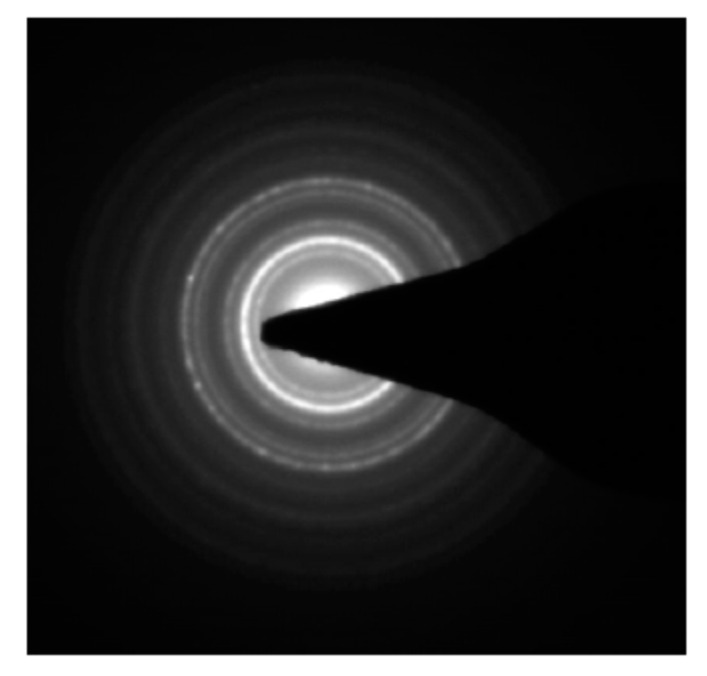
Electron diffraction study: Selected area electron diffraction (SAED) pattern of iron oxide nanoparticles showing their crystalline nature.

**Figure 4:**
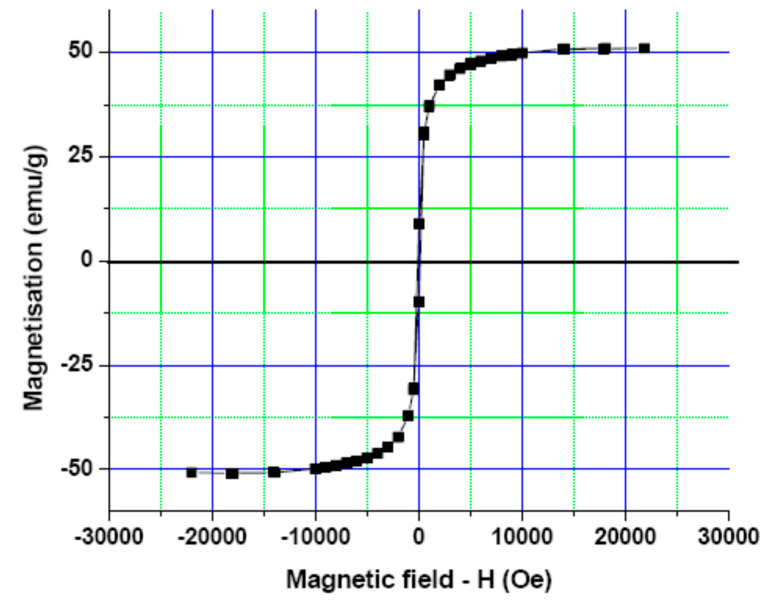
Vibrating sample magnetometer (VSM) analysis for magnetization: Magnetization curve obtained using VSM at 300 K (instrument temperature range: 2 to 1050 K) shows the super-paramagnetic nature of the synthesized iron oxide nanoparticles with a magnetization value of 50.9 emu/g.

**Figure 5:**
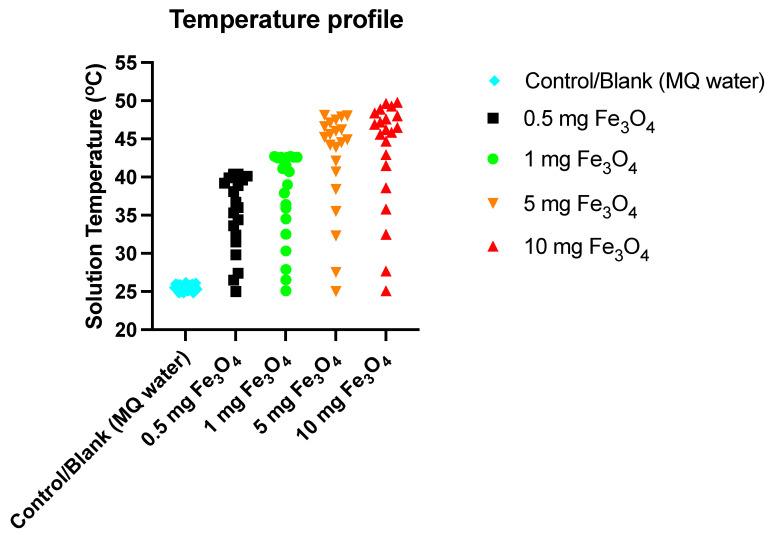
Photothermal/hyperthermia experiment: Temperature-dependent study—plot of rise in temperature vs. irradiation time (20 min) of the aqueous solutions of Fe_3_O_4_ nanoparticles with different concentrations. Irradiation was performed with a 635 nm red light laser.

**Figure 6:**
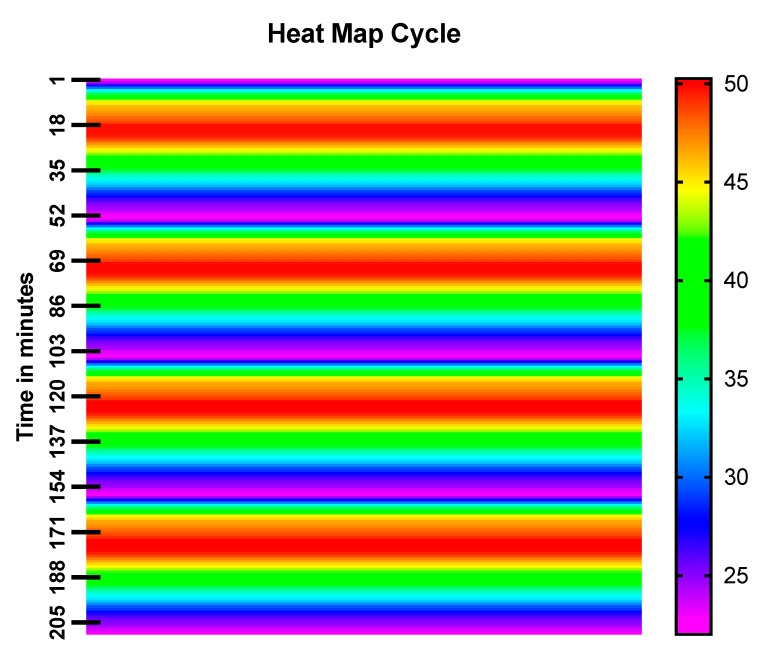
Heat map analysis: The graph represents back-to-back 04 repeated heat/cool cycles of SPIONs in the presence of 635 nm laser irradiation over a period of 210 minutes. The experimental analysis or measurement was performed at room temperature. Data represent the average of three individual experiments.

The co-precipitation method was used to develop an iron oxide nanoparticles (IONPs) under an inert atmosphere in aqueous medium. HRTEM and DLS techniques were used to determine the size of the formulation and the results revealed that the average particle diameter was around 15 nm (Figure 1a) and 25 nm (Figure 2a), respectively. The particles showed a narrow size distribution with spherical morphology and the surface charge/zeta potential was found to be negative (−29.6 mV; Figure 2b). The SAED pattern confirms the crystallinity of the synthesized particles (Figure 3). From the e-diffraction/SAED pattern, it is confirmed that crystals are oriented in different directions owing to the presence of concentric circles; therefore, the synthesized formulation is polycrystalline in nature. The d spacing value(s) of lattice planes (hkl) correspond to the JCPDS of iron oxide nanoparticles, thereby confirming the formation of magnetite (Fe_3_O_4_). In order to determine the magnetic properties of the synthesized iron oxide nanoparticles, VSM studies were performed, as shown in Figure 4, and the data/graph clearly depict their super-paramagnetic nature with a magnetization value of 50.9 emu/g. The particles were totally attracted by the magnet, as shown in Figure 1c. These nanoparticles were coated with a natural stabilizing agent; thus, the aqueous stability was then observed with pure tannic acid - a natural polyphenol. It was noticed that the aqueous dispersion of IONPs coated with tannic acid was stable for more than 72 h under normal conditions (RT) as compared with the uncoated formulation. The aqueous solution of coated nanoparticles with different amounts of Fe_3_O_4_ (0.5 mg, 1.0 mg, 5.0 mg, and 10.0 mg) was collected in a clear glass vial and irradiated with the visible red laser light (635 nm diode laser and a power density of 38 mWcm^−2^) for 20 min. The rise in temperature was monitored using a temperature thermocouple, which was inserted into the magnetic Fe_3_O_4_ solution. The graph of rise in temperature versus time was plotted as shown in Figure 5, and it was observed that there was a significant rise in temperature (~25 °C) within first 17 min of irradiation at the highest concentration, whereas the temperature of the control/blank (MQ water/PBS) showed an increase by approximately 4 °C in 60 min. To confirm the heat cycle efficacy, we performed a heat map experiment up to 04 ON/OFF cycles and measured the time-dependent change in temperature using a digital temperature thermocouple, as shown in Figure 6. From the results, it can be clearly seen that the particles maintain their hyperthermic property up to 04 cycles as we noticed.

**Figure 7:**
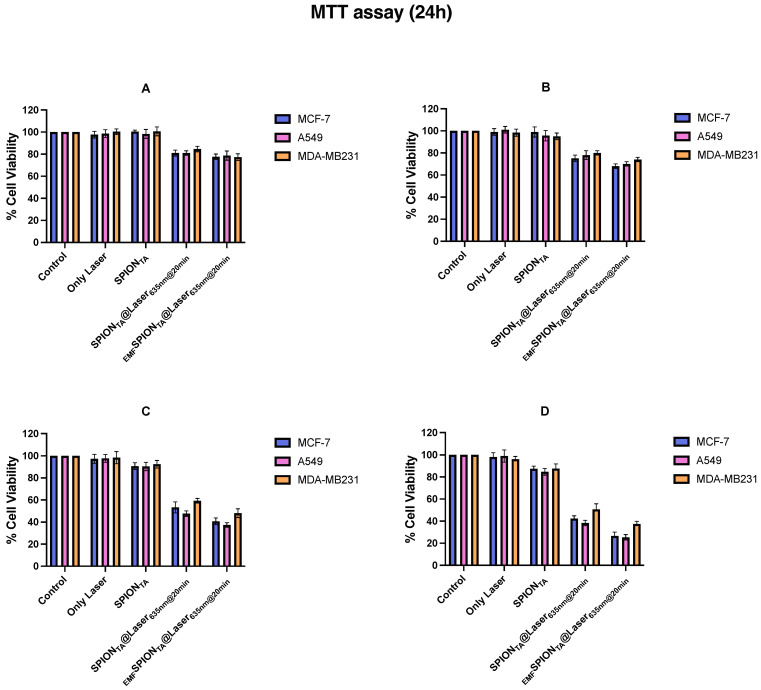
Cell viability assay (MTT assay): Cytotoxicity studies of iron oxide nanoparticles (pure SPIONs), laser, and SPIONs irradiated with a laser in the presence and absence of an applied external magnetic field (EMF) on MCF-7, A549, and MDA-MB231 cancer cell lines for 24 h at 37 °C. Analysis with different concentrations of stock solution ((**A**) (0.5 mg/mL), (**B**) (1 mg/mL), (**C**) (5 mg/mL), and (**D**) (10 mg/mL)) of iron oxide nanoparticles was carried out and then an appropriate concentration was established for further experiments. All experiments were performed in triplicate and the data were reported as mean ± SD.

**Figure 8:**
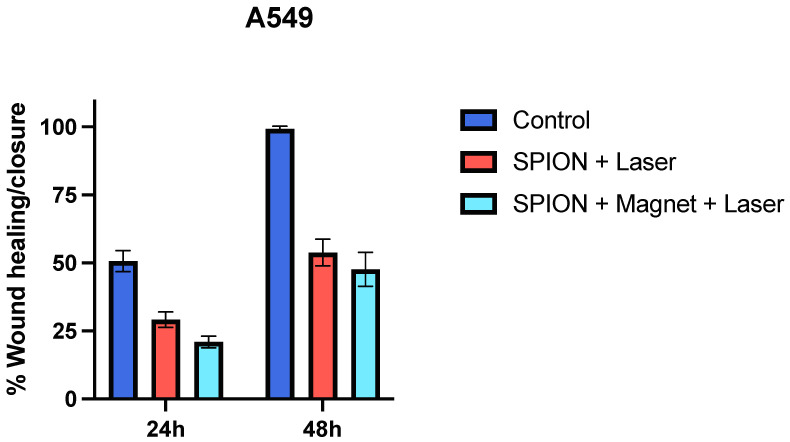
2-D Scratch assay: The data represent % healing/closure of wound/scratch compared with the control after 24 h and 48 h of treatment with SPIONs irradiated with a 635 nm laser in the presence and absence of an external magnetic field on lung cancer A549 cell lines at 37 °C. The analysis was performed with the help of ImageJ Software. Data represent mean ± SD of three individual experiments.

Next, we performed an *in-vitro* cell viability assay (MTT assay) to see if these superparamagnetic iron oxide nanoparticles (SPIONs) may be used as cytotoxin. We employed three different cancer cells for this assay: MCF-7, A549, and MDA-MB231. These cells were exposed to increasing concentrations of SPIONs and then the actual cytotoxic concentration was established for an experiment in combination with a laser or a magnetically guided SPIONs-laser system. After 20 min of irradiation, the plates were kept for 2 h inside the incubator, and then the treatment wells were replaced with fresh media and incubated for another 24 h at 37 °C before measuring the cell viability using MTT assay. We noticed considerable cell death in cancer cells treated with either the IONPs/laser or magnetically guided IONPs/laser combination (as shown in Figure 7). No significant cell killing was observed in the case of wells treated with either the laser or SPIONs alone. We also measured the total count of viable breast cancer cells (MCF-7) before and after the treatment and noticed a considerable decrease (nearly 2.3-fold) in the number of cells in the treated groups as compared with the control group after 24 h of incubation. Further, in order to evaluate the hyperthermia efficacy of the formulation on cell migration and invasion, a wound healing/scratch assay was performed. Cell migration, which involves the active movement of cancer cells from one area to another and relates to cancer metastasis, is regarded as a significant factor for any form of cancer. The 2D cell culture scratch assay analysis on A549 lung cancer cell lines showed that the cell migrates faster, and 100% wound/scratch closure was observed within less than 48 h in an untreated well/control, as compared with nearly 43% and 50% closure (almost two-fold inhibition) in the case of laser-irradiated nanoparticle-treated wells in the presence and absence of an external magnetic field, respectively. The results from the scratch assays, as seen in Figure 8, revealed that, in the presence of an external magnetic field, the nanoparticles had greater ability to inhibit cell migration in NSCLC cell lines. Real-time observation of the polythene bag (images not shown) clearly indicates that the irradiated dry powder/nanoparticles generate sufficient heat, which is responsible for damaging/rupturing the corner of the bag that is in direct contact of the powder, whereas the blank polythene bag was unaffected upon laser irradiation. Thus, the results predict that the ultra-small superparamagnetic iron oxide nanoparticles generate localized hyperthermia in tumor lesions, which could be an effective strategy for cancer therapy. Neither IONPs nor the laser alone resulted in any significant tumor cell killing, as shown by MTT assay. On the other hand, IONPs in the presence of a laser led to a significant decrease in cell viability with an optimum concentration of IONPs. From the data and the corresponding analysis, it is also depicted that cell viability is greatly hampered after the application of an external magnetic field. Therefore, this could be used as a tool for magnetically guided cancer therapy as well.

## 4. Conclusions

Super-paramagnetic iron oxide nanoparticles (SPIONs) were synthesized in aqueous medium under an inert (N_2_) atmosphere. The synthesized nanoparticles were characterized for their shape, size and surface charge using HRTEM, DLS and zetasizer. It was found that the size of the particles formed was around 15 nm, with almost spherical morphology and negative surface charge. Vibrating sample magnetometer (VSM) data confirm the super-paramagnetic nature of iron oxide nanoparticles. It was observed that the temperature of the solution containing nanoparticles reaches a maximum value of 49.8 °C within 17 min, whereas the temperature of the control/blank reaches a maximum value of 28.6 °C in 60 min. We further examined the magneto-photothermal strategy using iron oxide nanoparticles owing to their ability to be used as a possible treatment via magnetic hyperthermia. From 2D *in-vitro* cell culture results (cell viability measured using the MTT assay and wound healing analyzed using the scratch assay), it was observed that there was significant cell death in cancer cells incubated with the SPIONs/laser or magnetically guided SPIONs/laser combination. Moreover, cell death in the presence of an external magnetic field is comparatively more than in the absence of a magnetic field. Hence, in our report, we have summarized the synthesis of ultrasmall magnetic iron oxide nanoparticles and shown their dual nature to act as magnetically guided and photothermal agents for treatment against cancer.

## Data Availability

The data can be shared on request to the corresponding author after the ongoing studies have been completed and published.
